# An MRI Study of Morphology, Asymmetry, and Sex Differences of Inferior Precentral Sulcus

**DOI:** 10.1007/s10548-024-01035-5

**Published:** 2024-02-19

**Authors:** Xinran Zhao, Yu Wang, Xiaokang Wu, Shuwei Liu

**Affiliations:** 1 Department of Clinical Medicine, The Second Hospital, Cheeloo College of Medicine, Shandong University, Jinan, 250033 Shandong China; 2 Institute for Sectional Anatomy and Digital Human, Department of Anatomy and Neurobiology, Shandong Provincial Key Laboratory of Mental Disorder, Shandong Key Laboratory of Digital Human and Clinical Anatomy, School of Basic Medical Sciences, Cheeloo College of Medicine, Shandong University, Jinan, 250012 Shandong China; 3 Institute of Brain and Brain-Inspired Science, Shandong University, Jinan, 250012 Shandong China; 4 Department of Neurology, Tongji Medical College, Union Hospital, Huazhong University of Science and Technology, Wuhan, 430022 China

**Keywords:** Inferior precentral sulcus, Morphological patterns, Asymmetry, Sex disparity

## Abstract

Numerous studies utilizing magnetic resonance imaging (MRI) have observed sex and interhemispheric disparities in sulcal morphology, which could potentially underpin certain functional disparities in the human brain. Most of the existing research examines the precentral sulcus comprehensively, with a rare focus on its subsections. To explore the morphology, asymmetry, and sex disparities within the inferior precentral sulcus (IPCS), we acquired 3.0T magnetic resonance images from 92 right-handed Chinese adolescents. Brainvisa was used to reconstruct the IPCS structure and calculate its mean depth (MD). Based on the morphological patterns of IPCS, it was categorized into five distinct types. Additionally, we analyzed four different types of spatial relationships between IPCS and inferior frontal sulcus (IFS). There was a statistically significant sex disparity in the MD of IPCS, primarily observed in the right hemisphere. Females exhibited significantly greater asymmetry in the MD of IPCS compared to males. No statistically significant sex or hemispheric variations were identified in sulcal patterns. Our findings expand the comprehension of inconsistencies in sulcal structure, while also delivering an anatomical foundation for the study of related regions’ function.

## Introduction

**Fig. 1:**
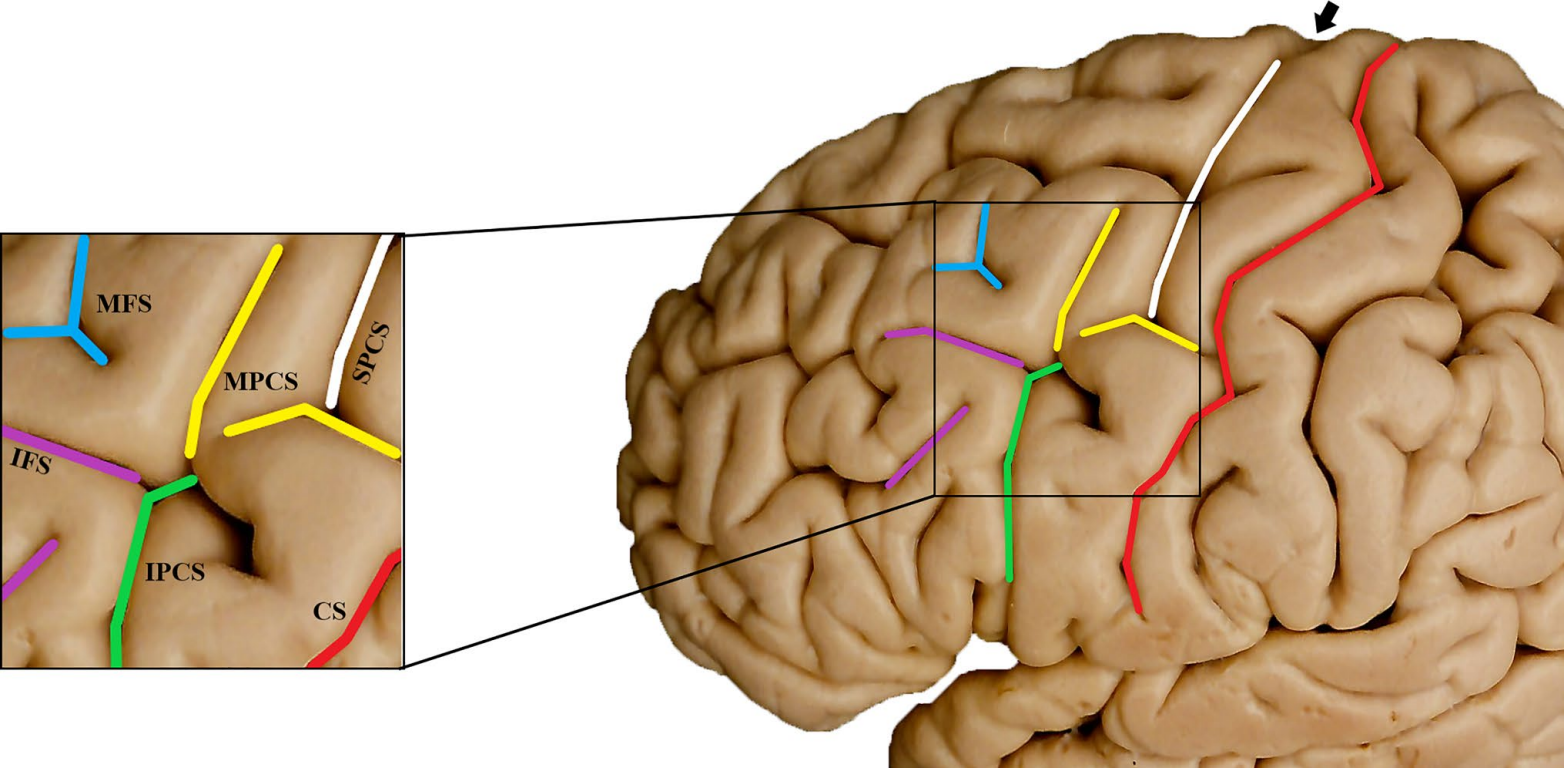
Lateral surface of the cerebral hemisphere. IPCS and its surrounding sulci are shown magnified in the black box on the left. Sulci in each picture are marked with different colors. IPCS, green, inferior precentral sulcus; IFS, purple, inferior frontal sulcus; CS, red, central sulcus; MPCS, yellow, intermediate precentral sulcus; SPCS, white, superior precentral sulcus; MFS, blue, intermediate frontal sulcus. The black arrow indicates the positions of the marginal precentral sulcus and the median precentral sulcus. The figure is based on human anatomic specimens

The precentral sulcus is located in the frontal lobe and is approximately parallel to the central sulcus. Its composition is intricate and called the precentral sulcal complex (Germann et al. 2005). As shown in Fig. 1, the composition of the precentral sulcal complex can be roughly described as follows: the intersection of the middle frontal gyrus and the precentral sulcus is the intermediate precentral sulcus (MPCS), the upper third of the precentral sulcus corresponds to the superior precentral sulcus (SPCS), the lower third represents the IPCS. Additionally, the marginal precentral sulcus and the median precentral sulcus are located along the upper border of the superior precentral sulcus. It is important to note that the aforementioned components may not be consistently present in every individual, suggesting potential individual variations in the IPCS.

The frontal cortex adjacent to the precentral sulcus can be classified into two regions: the supplementary motor cortex (SMC) and the premotor cortex (PMC). A systematic review has reported that the removal of brain tumors from the supplementary motor cortex may lead to a condition known as supplementary motor area syndrome (Palmisciano et al. 2022). Symptoms include severe motor dysfunction and language disorder, which indicates that the supplementary motor cortex may be responsible for planning, execution, and motor language. In a study utilizing functional magnetic resonance imaging (fMRI), Binkofski et al. observed neural activations in the premotor area when subjects were engaged in tasks involving the observation, imagination, and execution of finger movements(Binkofski et al. 2000). This study underscores the crucial role of the region surrounding the precentral sulcus in regulating language, sensation, and movement. In addition, it is noteworthy that numerous studies have demonstrated a consistent alignment between the location of neural activation and the sulcus (Malikovic et al. 2007; Rosano et al. 2002, 2003; Derrfuss et al. 2009). For instance, during task-switching, a neural activation that varied its location in accordance with the shape of the IPCS was observed at the intersection of the IFS and IPCS, known as the Inferior Frontal Junction (IFJ) (Derrfuss et al. 2009). Interestingly, we found that the IPCS is located at a key position that is closely related to these above cerebrocortical structures. Sulcal morphology as well as its variability are thought to affect the function of the relevant brain regions (Fedeli et al. 2020; Im and Grant 2019). Consequently, studying the morphological patterns of IPCS and its spatial relationships with IFS is of practical significance and will provide an anatomical basis for the functional studies of the related areas.

For decades, researchers have been fascinated by the individual variations in the complexity of cortical convolutions in the human brain. Recently, numerous studies have provided evidence of interhemispheric, sex, handedness, and race differences in the morphology and parameters of cerebral sulci (Wei et al. 2017; Wang et al. 2022; Tang et al. 2018). These differences are reflected in individual variations in brain cytoarchitecture and executive functions(Vogt et al. 1995; Fornito et al. 2004). Moreover, the influence of age and disease on the cerebral sulcus cannot be ignored (Raz et al. 2005; Zilles et al. 1988; Chaudhary et al. 2021; Kohli et al. 2019; Kim et al. 2008). There were significant differences between temporal lobe epilepsy patients and healthy people in the distribution pattern of sulci at the basal part of the temporal lobe (Kim et al. 2008). Abnormalities in sulcal morphology have even been detected in fetuses (Im and Grant 2019). For some specific diseases (such as Alzheimer’s disease), sulcal morphology has a supplementary diagnostic value (Bertoux et al. 2019).

Given the extensive focus on the functional aspects of the IPCS and its surrounding cortex, alongside the limited research on its structure, we have chosen IPCS as our research subject. This will deepen our understanding of the morphological asymmetry of brain sulci and sex dimorphism. Furthermore, it holds the potential to establish a relationship between the structure and function of IPCS in the future. We used brainvisa-5.0.2 software to analyze a large sample of in vivo brain MR images, performed the three-dimensional reconstruction, and calculated the morphological parameters. A key point is that our study uses a neuroimaging method instead of traditional autopsies, which has a wider sample source, quantitative measurements, and makes more sense in clinical practice.

## Materials and Methods

### Subjects

A total of 92 Chinese volunteers were recruited for this study, including 54 males (mean age 17.11 ± 1.35 years) and 38 females (mean age 17.45 ± 1.76 years). All subjects underwent clinical, laboratory, and imaging examinations to confirm their good health and absence of neuropsychiatric diseases. According to the Edinburgh handedness standard test results, all subjects were right-handed. The study obtained experimentally informed consent from all participants and their parents, and it was approved by the Ethics Committee of Shandong University Medical College.

### Data Acquisition

The MR images were acquired using a 3.0T GE SIGNA magnetic resonance scanner (GE Medical System, Milwaukee, USA). The scanning baseline was AC-PC line, and the scanning orientation was axial position. The scanning sequence was T1-weighted fast spoiled gradient-echo (FSPGR). Scanning parameters: repeat time(TR) 6.8ms, echo time(TE) 2.9ms, flip angle 10°, number of excitations(NEX) 2, scanning layers 248, scanning time 12 min, field of view (FOV) 24 cm×24 cm, voxel size 0.47 mm×0.47 mm×0.47 mm.

### Image Processing

**Fig. 2:**
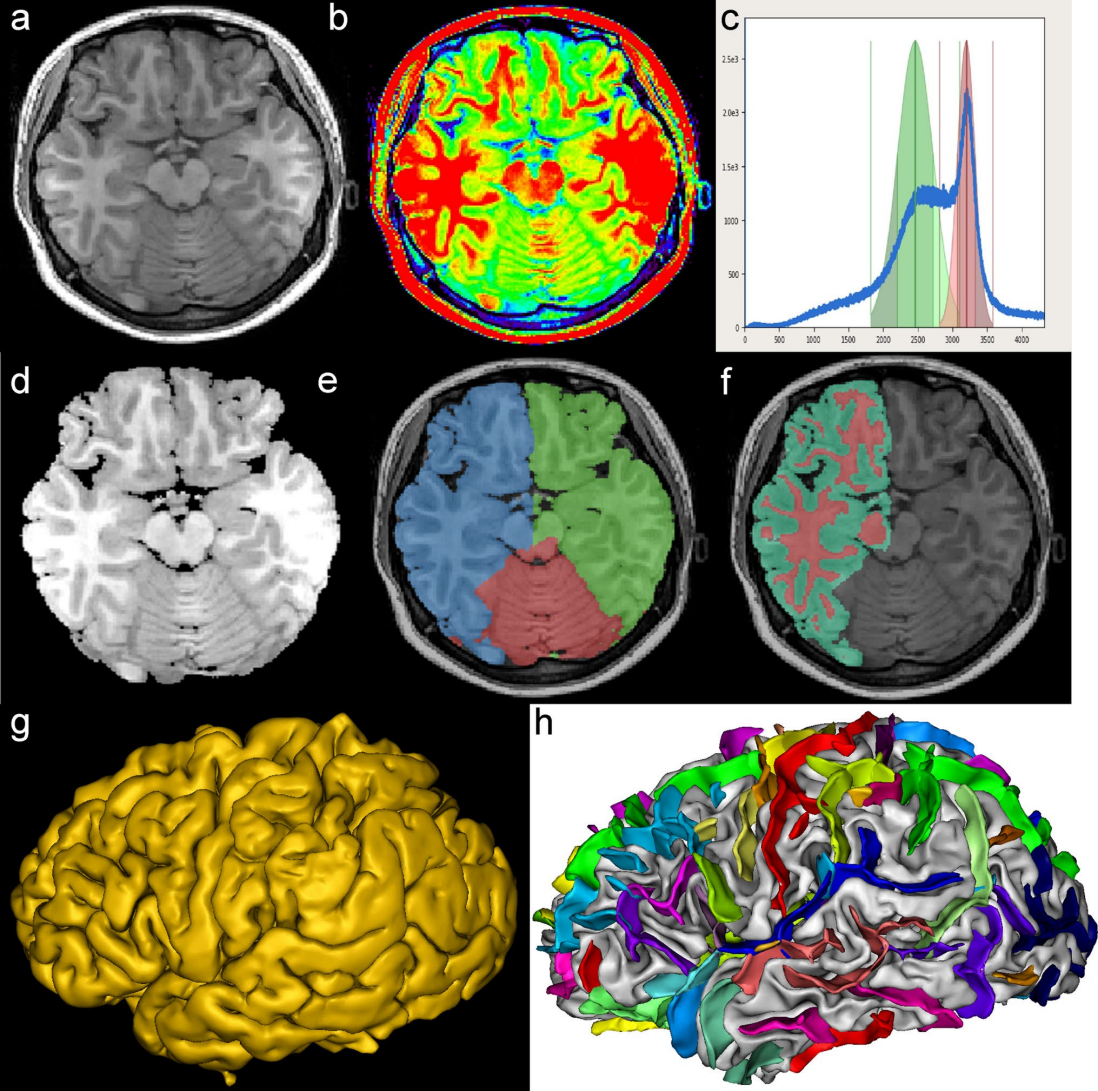
Brain surface reconstruction, sulci extraction, and recognition. **a** Raw TI image; **b** bias correction; **c** histogram analysis (green, red, and blue lines indicate GM, WM, and CSF respectively); **d** Removal of non-brain tissue; **e** Calculating brain mask; **f** Brain tissue is segmented into GM and WM. This step is carried out in hemispheres. **g** Reconstruction of brain surface; **h** Sulci extraction and recognition. Each picture is illustrated by snapshots of the corresponding process

The MR images were processed using Brainvisa-5.0.2 software (https://brainvisa.info/web/). First, the obtained T1 phase MR images were imported into Brainvisa software. Second, we manually identified the anterior commissure (AC), posterior commissure (PC), and the midpoint between the left and right hemispheres in the image in order to prepare for registration to Talairach space. Third, the object-based morphology pipeline of the software was initiated to automatically process the data. This pipeline encompassed several steps including bias correction, histogram analysis, non-brain tissue removal, segmentation, gray matter (GM)and white matter (WM) reconstruction, brain sulci extraction and recognition, and parameters measurement (Fig. 2). Bias correction was performed to mitigate the influence of field inhomogeneity as demonstrated in Fig. 2b. In the histogram analysis, Fig. 2c illustrates distinct peaks for GM, WM, and cerebrospinal fluid (CSF), reflecting the number of voxels in different brain components, so as to prepare for the brain segmentation. Subsequently, non-brain tissue was removed and a brain mask was generated using the information acquired from the above steps (Fig. 2d,e). Afterward, brain tissue was segmented into GM and WM (Fig. 2f). These two parts were merged to create the outer surface of the brain as shown in Fig. 2g. Finally, each sulcus was recognized and labeled with different colors (Fig. 2h).

Extracting and recognizing brain sulci is challenging due to their complex morphological variability. To address this problem, the software uses a sulcal root recognition strategy. The first part of cortical folding during the embryonic formation of each brain sulcus is called the sulcal root, which exhibits relatively stable morphology across different individuals (Regis et al. 2005). The software implements the SPAM algorithm to assign at most one 3D label to each voxel based on the probability of it belonging to a particular brain sulcus. Voxels with the same label are then combined to form a specific brain sulcus (Perrot et al. 2009, 2011). The software incorporates the Bayesian framework to enhance the accuracy of recognizing small branches adjacent to the brain sulcus. The overall recognition accuracy for sulci reached 86% (Perrot et al. 2009). In anatomical studies, the use of software instead of manual methods is necessary because the data on sulcal morphology is relatively complex and not completely understood. Therefore, it is not possible to pre-configure a sulcal model and it is necessary to empirically estimate it from a training database. In general, we believe that the Brainvisa software provides a more reliable judgment on the attribution of small branches of the cerebral sulcus compared to manual labeling. Therefore, we no longer perform manual corrections in this aspect. However, we have made corrections to some obvious errors through our examination. In order to minimize subjective factors in sulcus classification, all the authors have reached a consensus on the classification and referred to Ono et al.’s cerebral sulcus atlas (Ono et al. 1990), Brainvisa’s cerebral sulcus atlas(https://brainvisa.info/web/), and correctly identified cerebral sulcus images.

**Fig. 3:**
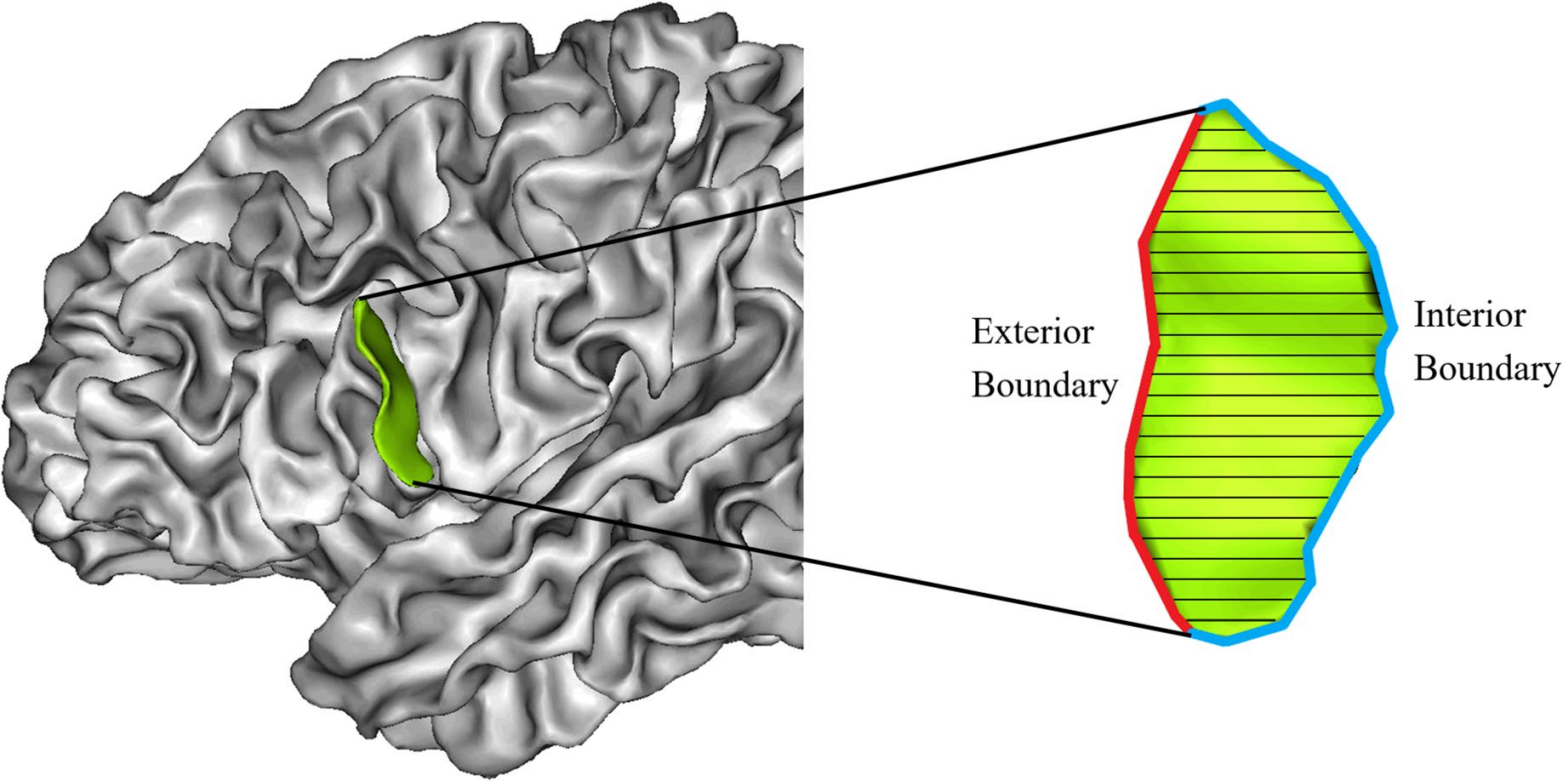
Methods of gauging MD is shown in the figure. The exterior boundary is marked red, the interior boundary is marked blue and black lines represent the measuring lines

The mean depth (MD) algorithm was developed by Kochunov (Kochunov et al. 2012). Depth is defined as the projection of a line that connects the exterior boundary (top ridge) and interior boundary (fundus) of a sulcus. The top ridge and fundus were subdivided into 100 equidistant intervals to form 100 measuring lines. The average value of the 100 measured depths is called the MD (Fig. 3). To enable inter-individual comparison of MD, registration is a prerequisite step. Brainvisa’s registration process incorporates Talairach registration, global registration, and local registration. Talairach registration method aligns the image to the Talairach atlas by aligning the AC-PC line. Global registration is utilized for the overall alignment of brain sulci, whereas local registration, building upon global registration, further refines the alignment of each sulcus (Perrot et al. 2011).

### Statistical Analysis

The Chi-square test was used to analyze the significance of interhemispheric and sex differences in morphological patterns of IPCS. Covariance analysis and T-tests were conducted to examine the variation in MD of IPCS across hemispheres and sex. Age and brain surface area were incorporated as covariates in the covariance analysis to account for the potential impact of these variables on the model. *P* < 0.05 was considered statistically significant. All statistical analyses were performed by IBM SPSS 25.0 software.

## Results

A total of 92 subjects were counted, including 54 males and 38 females. IPCS was observed in 172 hemispheres, accounting for 93.5% (90 in the left hemisphere and 82 in the right hemisphere). A total of 12 cases (6.5%) were lack of IPCS (2 cases in the left hemisphere and 10 cases in the right hemisphere).

### Morphological Patterns of IPCS

**Fig. 4:**
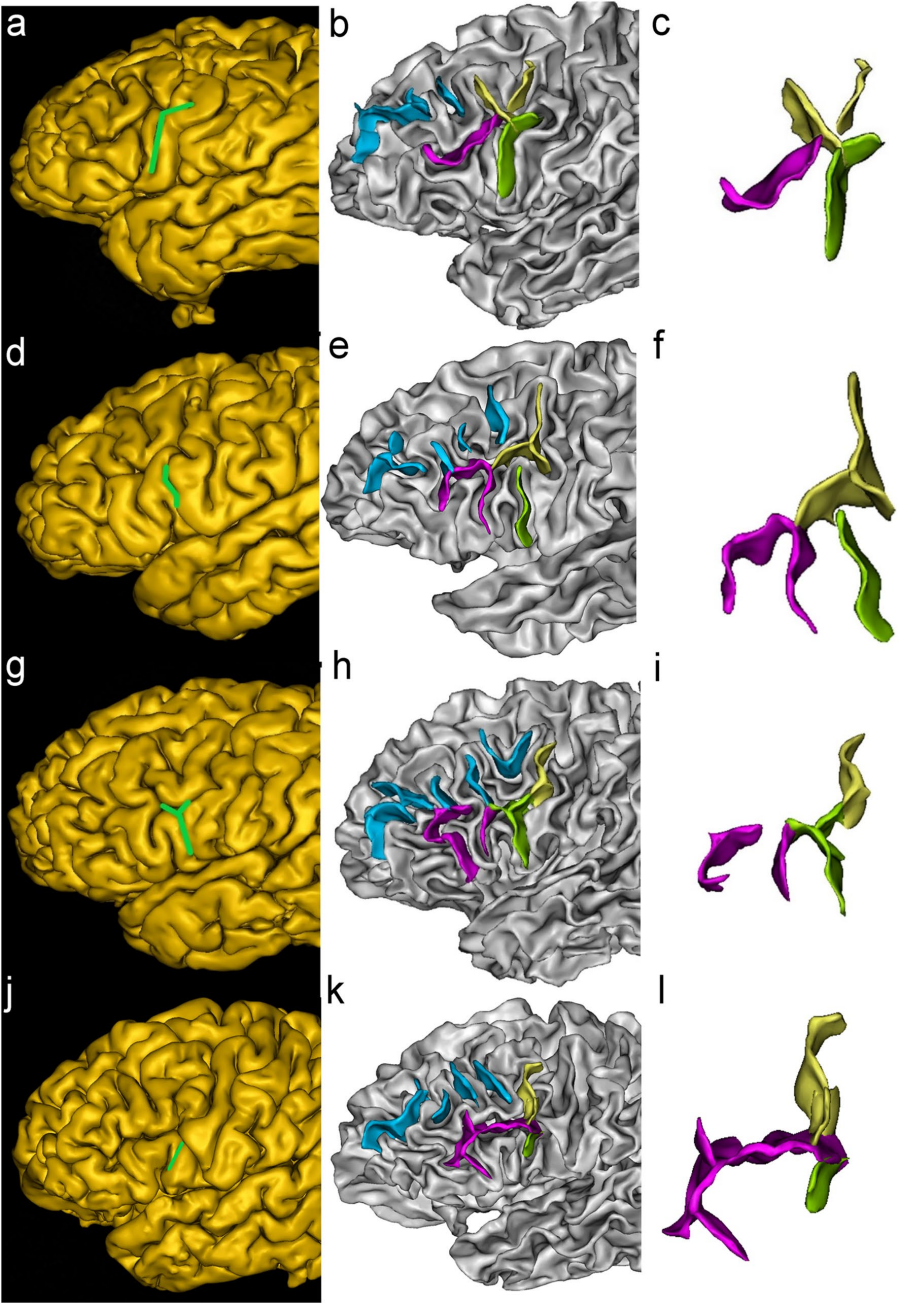
Examples of IPCS morphological patterns. Sulci in each picture are marked with different colors: IPCS: green; IFS: purple; MPCS: yellow; MFS: blue. **a–c**: arcuate from; **d–f**: bayonet form; **g–i**: Y form; **j–l**: tiny form. Each picture is illustrated by snapshots of sulci extraction and sulci details

IPCS could be categorized into five types: arcuate form, bayonet form, Y form, tiny form, and ramified form (Fig. 4).

#### Arcuate form

The arcuate form consists of two parts: a trunk that runs in a dorsoventral direction and a branch running along the rostrocaudal side originating from the trunk’s dorsal end (Consistent with this type, the trunk of other types has basically the same track). A total of 58 hemispheres were observed, accounting for 33.7%.

#### Bayonet form

The bayonet form has only one curved trunk and contains no branches. This is the most common type, with 75 hemispheres, accounting for 43.6%.

#### Y form

The Y form contains one trunk and two branches originating from its dorsal end. The trunk and two branches are arranged in a letter “Y” shape, with 7 hemispheres, accounting for 4.1%.

#### Tiny form

The tiny form has only one trunk, relatively small and straight, with 11 hemispheres, accounting for 6.4%.

#### Ramified form

**Fig. 5:**
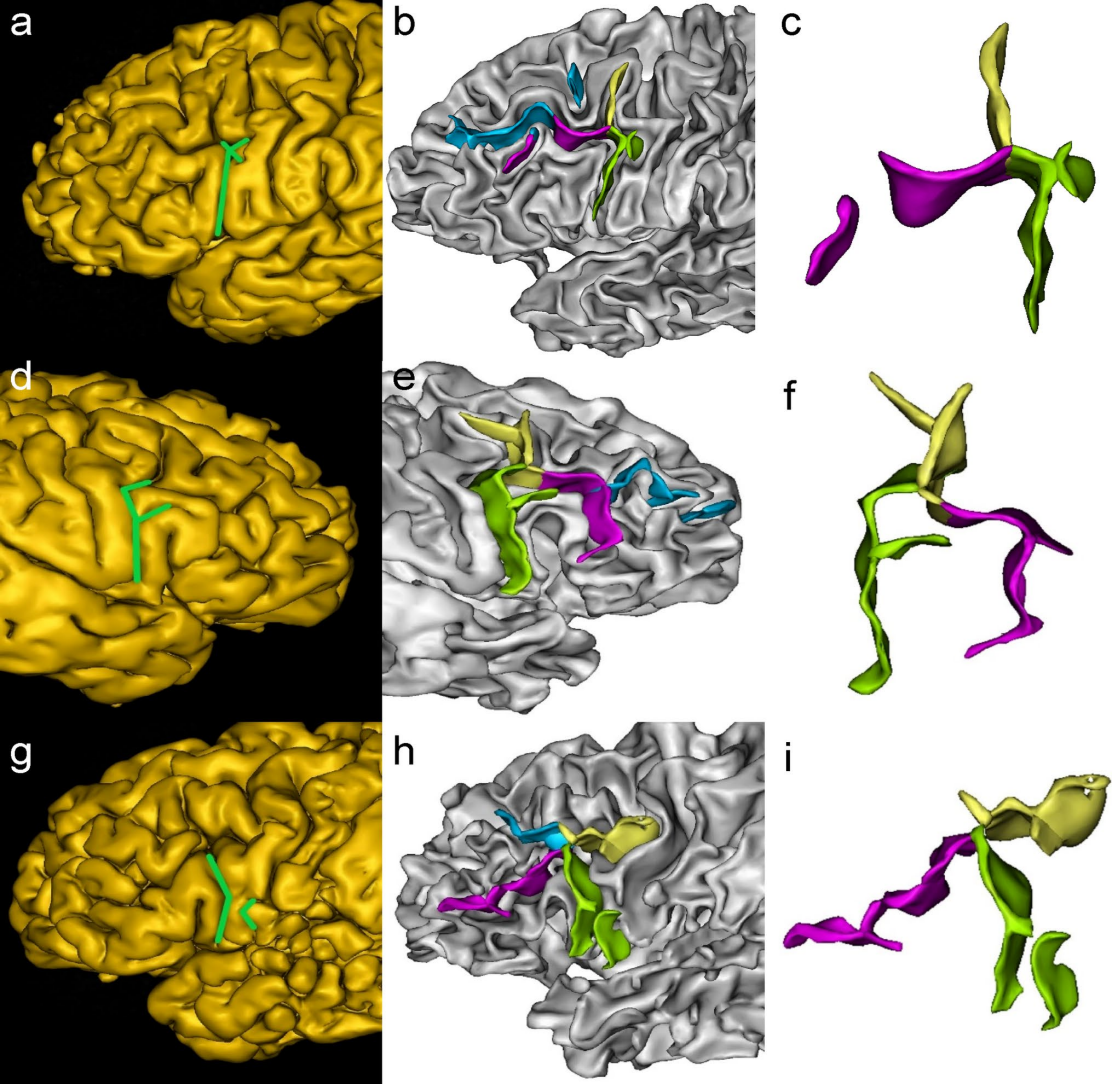
Examples of IPCS ramified form. The color labeling of sulci is the same as in Fig. 4**a**–**c**: The second branch starts from the bottom of the first branch; **d–f**: The second branch starts from the middle of the trunk; **g–i**: The second branch is completely separate from the trunk and the first branch. Each picture is illustrated by snapshots of sulci extraction and sulci details

The ramified form consists of three parts: one trunk and two branches. The trunk and the first branch run basically the same as the arcuate form. In addition, there is a second branch originating from the trunk or the first branch of IPCS. There is also a special type in which the second branch is completely separate from the trunk and the first branch. A total of 21 hemispheres were observed, accounting for 12.2% (Fig. 5).

### Interhemispheric Differences in Morphological Patterns

Overall, the distribution of IPCS sulcal patterns is similar in both the left and right hemispheres. The majority of sulci are in a bayonet form, followed by arcuate form and ramified form. Y form and tiny form are less common. (Table 1)

However, there was no statistically significant interhemispheric difference in all morphological types (χ^2^ = 1.028, *P* = 0.905) and each type respectively. The incidence of the Y form and tiny form is too small to be analyzed statistically. The results of the Chi-square test are shown in Table 1.

**Table 1:** Frequency and percentage of morphological patterns in IPCS (classified by hemispheric side)

	Bilateral hemispheres(numbers/proportion)	Left hemisphere(numbers/proportion)	Right hemisphere(numbers/proportion)	χ^2^	P
Arcuate form	58/33.7%	33/36.7%	25/30.5%	0.733	0.392
Ramified form	21/12.2%	11/12.2%	10/12.2%	0.000	0.996
Bayonet form	75/43.6%	37/41.1%	38/46.3%	0.477	0.490
Y form	7/4.1%	4/4.4%	3/3.7%	–	–
Tiny form	11/6.4%	5/5.6%	6/7.3%	–	–

### Sex Differences in Morphological Patterns

Similarly, the morphological distribution of IPCS is predominantly bayonet form for both males and females, followed by arcuate form and ramified form. Y form and tiny form are relatively less common. (Table 2)

No statistically significant between-sex difference was detected for all (χ^2^ = 1.566, *P* = 0.815) and each morphological type. The results of the Chi-square test are shown in Table 2.

**Table 2:** Frequency and percentage of morphological patterns in IPCS (classified by sex)

Morphological patterns	Hemisphere	Sex		χ^2^	P
		male	female		
Arcuate form	left	17	16	0.210	0.647
	right	16	9		
Ramified form	left	7	4	0.538	0.463
	right	7	3		
Bayonet form	left	24	13	0.214	0.644
	right	19	19		
Y form	left	2	2	–	–
	right	2	1		
Tiny form	left	5	0	–	–
	right	3	3		

### Spatial Relationship between IPCS and IFS

**Fig. 6:**
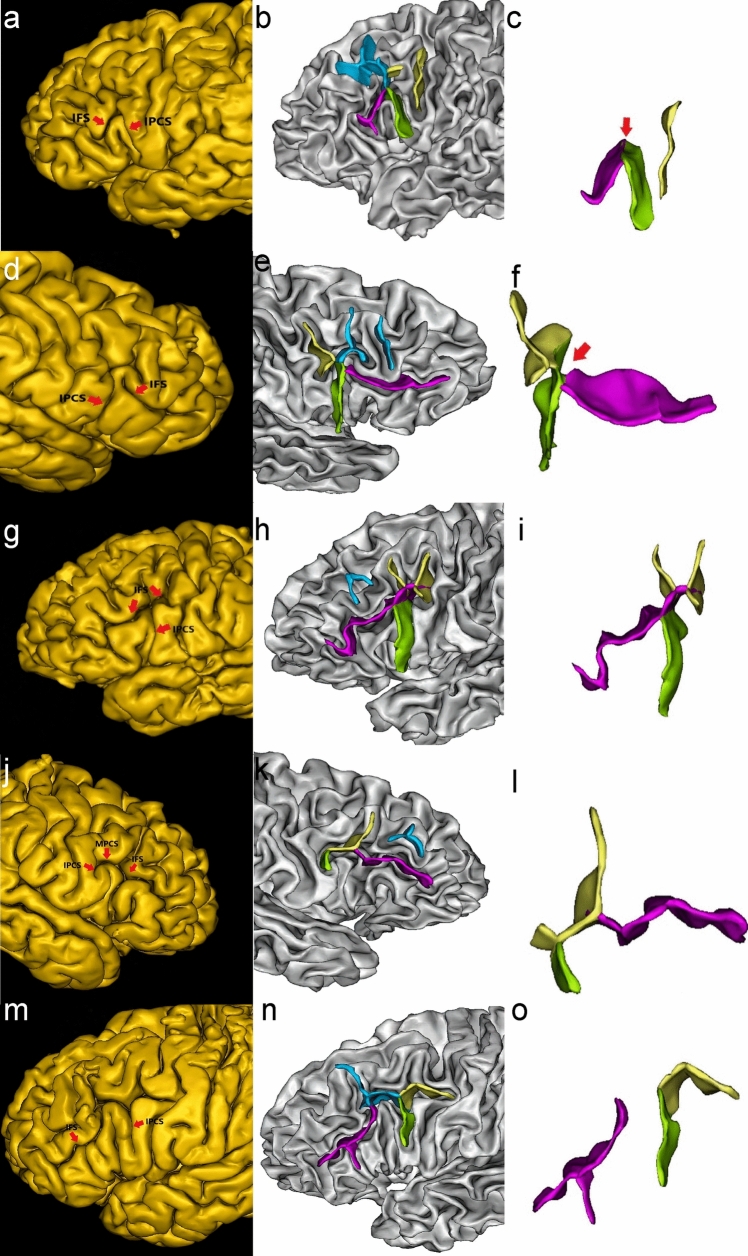
Examples of the spatial relationship between IPCS and IFS. **a–c**: True connection form, the red arrow in picture **c** shows IPCS and IFS are completely connected; **d–f**: Pseudoconnection form, the red arrow in picture f shows IPCS and IFS are connected superficially; **g–i**: Through-connection form; **j–l**: IFS are connected with the MPCS; **m–o**: IFS is completely separated from the precentral sulcus. Each picture is illustrated by snapshots of sulci extraction and sulci details.

The spatial relationship between IPCS and IFS could be categorized into four types: true connection form, pseudoconnection form, no connection form, and through-connection form (Fig. 6). The number and proportion of each type are shown in Table 3.

**Table 3:** Frequency and percentage of spatial relationship between IPCS and IFS

Spatial relationship		Bilateral hemispheres(numbers/proportion)	Left hemisphere(numbers/proportion)	Right hemisphere(numbers/proportion)
True connection form		89/51.7%	51/56.7%	38/46.3%
Pseudoconnection form		33/19.2%	13/14.4%	20/24.4%
Through-connection form		4/2.3%	4/4.4%	0/0.0%
No connection form	Completely separate	32/18.6%	15/16.7%	17/20.7%
	Connection with MPCS	14/8.1%	7/7.8%	7/8.5%

#### True Connection form

IPCS and IFS are completely connected in the superficial and deep layers of the cerebral cortex. This is the most common type in the spatial relationship between IPCS and IFS. (89 hemispheres, 51.7%)

#### Pseudoconnection form

IPCS and IFS appear to be connected on the surface of the cerebral cortex but are deeply separated from each other due to interruption by gyri deep in the intersection. (33 hemispheres, 19.2%)

#### Through-connection form

The rostral end of IFS runs through IPCS. This is the rarest type (4 hemispheres, 2.3%).

#### No Connection form

There is no connection between IPCS and IFS. Some of the IFS are connected with the MPCS, while the others are completely separate from the precentral sulcus.

### Interhemispheric Differences in mean Depth

After including brain surface area and age as covariates in the model, the analysis results showed a significant main effect of brain surface area (F = 14.038, *P* < 0.001), while the main effect of age was not significant (F = 0.537, *P* = 0.465). The interhemispheric difference in MD of IPCS was not statistically significant (F = 2.062, *P* = 0.153) after controlling for the confounding effects of these two covariates (Table 4). Furthermore, we conducted a T-test to study interhemispheric differences in males and females respectively, results are shown in Table 5. Interestingly, we found that females had significantly higher asymmetry in MD of IPCS than males.

**Table 4:** Results of covariance analysis for MD of IPCS

Grope	Hemisphere	Sex		Interhemispheric		Sex		Hemisphere×sex	
MD(mm)		Male	Female	F	P	F	P	F	P
	Left	16.13±2.94	16.46±3.83	2.062	0.153	14.388	＜0.001	3.614	0.059
	Right	16.01±4.55	18.27±2.74						
	Bilateral	16.07±3.77	17.34±3.44						

The descriptive values are shown in mean ± SD

**Table 5:** Results of T-test for MD of IPCS

**Group**	**Value1**	**Value2**	**T**	**P**
I	16.13 ± 2.94	16.01 ± 4.55	0.147	0.884
II	16.46 ± 3.83	18.27 ± 2.74	-2.283	0.026^*^
III	16.13 ± 2.94	16.46 ± 3.83	0.439	0.662
IV	16.01 ± 4.55	18.27 ± 2.74	2.789	0.007^*^

Group I: interhemispheric difference of MD in males; Group II: interhemispheric difference of MD in females; Group III: sex difference of MD in the left hemisphere; Group IV: sex difference of MD in the right hemisphere. In Group I and II Value1 represents the MD of the left hemisphere; Value2 represents the MD of the right hemisphere. In Group III and IV, Value1 represents the MD of males; Value2 represents the MD of females. Asterisk (*) indicates that p<0.05; The descriptive values are shown in mean ± SD

### Sex Differences in mean Depth

As shown in Tables 4 and 5, after controlling for the influences of age and brain surface area on MD, it was observed that females demonstrated a significantly deeper IPCS compared to males, with this distinction primarily localized in the right hemisphere. No significant interaction between hemisphere and sex was found in MD. In addition, the MD of IPCS is deeper in females compared to males in left, right, and bilateral hemispheres.

## Discussion

### Morphological Patterns and Functions of IPCS

In the atlas we referred to (Ono et al. 1990), the author conducted dissections on 25 human brains to examine the morphological structures of IPCS. Four distinct forms were identified: arcuate form, ramified form, bayonet form, and Y form, representing 38%, 32%, 24%, and 6% of the total occurrences, respectively. Compared to our study, the proportion of arcuate form and Y form is similar, while it has a higher proportion of ramified form and a lower proportion of bayonet form. We consider that the possible reason for the discrepancy is the different data processing methodologies. The data in the atlas is derived from postmortem brain anatomy and direct observation, this method can detect small branches, but it is more subjective. The data in our study are derived from MR image analysis of living brains. The advantage is that there are unified standards for the extraction and recognition of brain sulci, but image distortion is inevitable. A study comparing different brain-mapping techniques showed that the SPAM model of Brainvisa had a relatively lower validity for recognizing small branches of brain sulci (Perrot et al. 2011). Taking the fact ramified form has the most branches and the bayonet form has the least into consideration, we can speculate that Brainvisa is easier to recognize brain sulci with fewer branches, which may lead to this discrepancy. Tiny form was not reported in the atlas, possibly due to the limited sample size and the challenges associated with manual identification of the tiny form sulcus. Other studies have also reported on the morphology of IPCS, but their classification methods were relatively simple (Germann et al. 2005; Ebeling et al. 1989). For instance, Germann et al. provided a description of the horizontal branch of the IPCS (Germann et al. 2005).

With regard to spatial relationships, IPCS was connected with the precentral sulcus in 81.4% of subjects. Some studies have reported similar results (Ribas et al. 2006; Ebeling et al. 1989). We use the “sulcal pit” theory to explain this phenomenon (Welker 1990). The rapidly-growing brain region during embryonic development forms gyri, and the margins of these gyri form sulcal pits which are the locally deepest region of the sulci and are believed to have a close relationship with brain function (Lohmann et al. 2008; Welker 1990). For existing studies on some sulci, a sulcal pit is located at IFJ (Im et al. 2010). Thus, it can be inferred that in the pseudoconnection form, the deep gyri within the intersection exist as landmarks for separating sulci, while the surrounding areas represent the locations of sulcal pits. During embryonic development, if the gyri in the intersection were completely merged with the peripheral sulci, the True connection is formed; if it is not merged at all, then the No connection is formed. Over the past few decades, the function of IFJ has been widely investigated and proved to be potentially important (Derrfuss et al. 2009, 2012; Tamber-Rosenau et al. 2018; Cole and Schneider 2007; Roth et al. 2006; Ekert et al. 2021; Ruland et al. 2022), which can provide additional evidence for the above explanation.

The exact mechanism by which cortical folding occurs remains unclear. In 1945, Le Gros Clark first proposed the skull constraint theory (Clark 1945). To date, significant progress has been made in research on the mechanical tension model, suggesting that the deformation of the cerebral cortex under axonal tension contributes to the formation of cerebral convolutions (Hilgetag and Barbas 2006; VanEssen 1997). The mechanical properties of white matter fibers and cortical thickness also play a pivotal role in this process (Toro and Burnod 2005). In addition, different cytoarchitectures are believed to cause differential expansion of the cortex, leading to distinct superficial topographies (Ronan et al. 2014). Gene expression, environmental factors, sex hormones, craniocerebral injury, etc., are thought to regulate or influence this process (Chi and Chun 1941; Miller et al. 2014; VanEssen 1997; Ristori et al. 2020). The precentral sulcus emerges around the 24th week of gestation and is roughly parallel to the central sulcus(Chi et al. 1977). According to the order in which brain sulci appear during embryonic development, scientists classify them as primary (before 32w of gestation) secondary (gestation 32w-36w), and tertiary sulci (later than 36w gestation) (Chi et al. 1977; Yao et al. 2023; Miller et al. 2021). The primary sulci are deeper and more genetically stable, whereas tertiary sulci are shallower, more susceptible to non-genetic factors, and show variability (Lohmann et al. 1999). Accordingly, the IPCS (mean depth 16.6 mm, appearing at 24 w of gestation) belongs to the primary sulcus. Kruggel and colleagues suggest that the primary sulcus discussed here is part of the core sulcal region, which exhibits the most abundant fiber connections within and between brain regions, occupying a central role in interregional communication (Kruggel and Solodkin 2023). Parameters that reflect the brain’s anatomical structure, such as sulcal depth and cortical thickness, have the potential to influence the formation of these brain connections and consequently impact brain function. A neuroanatomical study conducted on macaque monkeys revealed the presence of short-range white matter fibers originating from the bottom of the sulcus, facilitating communication between neighboring brain regions. Deeper sulci imply the existence of shorter white matter fibers, resulting in enhanced efficiency of neural processing (Reveley et al. 2015; Voorhies et al. 2021). The Sulcal pattern is established during embryonic development and remains stable throughout the developmental period from childhood to adulthood. However, the quantitative anatomical measurements of sulci undergo changes throughout adulthood (Tissier et al. 2018; Cachia et al. 2016). Multiple studies have indicated a correlation between sulcal patterns and various brain functions (Cachia et al. 2016, 2018; Tissier et al. 2018; Borst et al. 2016). For example, Tissier et al. found that sulcal patterns of the dorsal anterior cingulate cortex and the inferior frontal cortex impact inhibitory control efficiency in children. These disparities in inhibitory control efficiency originate during the fetal stage and coincide with the developmental period of gyrification patterns (Tissier et al. 2018). Children can enhance inhibitory control efficiency through training, a process known as neural plasticity. However, sulcal patterns remain stable throughout this process, potentially limiting the effectiveness of acquired training (Cachia et al. 2016). Recently, Pang et al. presented a model that explores the cortical and subcortical activity triggered by resonant modes of the brain’s geometry. This model, different from the classical theory of brain connectivity, provides us with a novel mechanism by which the geometric shape of the brain governs its functional characteristics (Pang et al. 2023).

The functions of the IPCS and its surrounding areas are diverse, including visuospatial attention (Tamber-Rosenau et al. 2018), cognitive control (Cole and Schneider 2007), task switching (Derrfuss et al. 2009), working memory (Roth et al. 2006) and pronunciation(Flinker et al. 2015). Recent research has focused on elucidating the specific functions of distinct regions within the IPCS. In their study, Ekert et al. classified functional areas adjacent to the IPCS into three parts: the ventral precentral gyrus bordering the precentral sulcus(vpcg/vpcs), the dorsal precentral gyrus(dpcg), and IFJ.They proposed that vpcg/vpcs was involved in the sublexical assembly of articulatory plans, dpcg was associated with word retrieval and IFJ was implicated in cognitive control and working memory (Ekert et al. 2021). Similarly, Ruland et al. have categorized IFJ into two parts, IFJ1 and IFJ2, based on differences in cytoarchitecture and receptor types. They suggested that the variable sulcal pattern of IFJ contributed to the variability in the localization of IFJ1 and IFJ2 (Ruland et al. 2022). Consequently, we hypothesize that the spatial relationship between IFS and IPCS will contribute to inter-individual variability in cognitive and behavioral functions, necessitating further investigation.

### Interhemispheric Differences of IPCS

Language function of brain exhibits a significant degree of lateralization, with a prominent dominance of left hemisphere. Research indicates that this phenomenon is observed in 96% of right-handed individuals and 73% of left-handed individuals(Knecht et al. 2000). In contrast, music processing and attention show right lateralization (Ruland et al. 2022). Numerous studies have proposed a correlation between brain anatomical structures and the lateralization of functions. Foundas et al. (1996) observed that 9 out of 10 individuals with left language lateralization exhibited significant left asymmetry in the Pars triangularis of Broca’s area, characterized by a greater surface area in the left hemisphere (Foundas et al. 1996). Moreover, significant asymmetry was observed in Heschl’s gyrus, the lateral fissure, and the superior temporal sulcus (Good et al. 2001; Ochiai et al. 2004). Furthermore, there is a correlation between the degree of asymmetry and sex, wherein interhemispheric differences are significantly greater in males than in females (Kulynych et al. 1994; Amunts et al. 2000; Bear et al. 1986). Likewise, male language function tends to be more lateralized, while female language function tends to be more bilateral. This was exemplified in a study conducted by Amunts et al., revealing that in right-handed males, the depth of the left central sulcus was significantly greater than that in the right hemisphere, whereas it was not significant in females (Amunts et al. 2000).

In our study, although no interhemispheric statistically significant differences were found in MD and morphological patterns of IPCS, females showed a significant rightward asymmetry compared to males in MD. Contrary to the above mentioned, the possible reason is different ages. Amunts’s study (Amunts et al. 2000) had a larger age range (17–49 years for males, 15–55 years for females), while our study recruited younger participants with a narrower age range. Our hypothesis may be supported by a study by Jao et al., which indicated that young females displayed greater cortical lateralization compared to males. With age progressing, males underwent increasing cortical lateralization (Jao et al. 2021).

### Sex Differences of IPCS

Brain differences between males and females are both structural and functional. In terms of structure, adult males exhibit significantly larger total brain volume, brain weight, and brain surface area compared to females overall. On the other hand, adult females are characterized by a thicker and denser cortex (Raz et al. 1997; Cosgrove et al. 2007; Ritchie et al. 2018; Luders et al. 2005). However, these differences are not uniform across all brain regions. For example, males tend to have a thicker right insula compared to females (Ritchie et al. 2018). Age plays a crucial role in brain differences between males and females, as specific sex differences vary at different stages of development due to distinct developmental trajectories (Kaczkurkin et al. 2019). Findings from a longitudinal study indicated that although adult males generally have larger cortical surface areas than females, females reached their peak earlier than males (8.1 years for females and 9.7 years for males) (Raznahan et al. 2011).

Our study showed a statistically significant sex difference in MD of IPCS. Females exhibited significantly deeper MD in IPCS compared to males, predominantly in the right hemisphere. This finding aligns with a study that showed the global sulcal index(g-SI) was significantly higher in females compared to males and the right hemisphere compared to the left (Liu et al. 2010). G-SI is defined as the ratio between the total sulcal area and the outer cortex area. The higher g-SI observed in females is attributed to various regional sulcal parameters, including MD. Another relevant study by Luders et al. (2004) observed that females exhibited greater gyrification in the frontal and parietal regions compared to males, further supporting the abovementioned view (Luders et al. 2004). One unresolved issue pertains to the influence of parameters such as the depth and length of a certain sulcus on the overall gyrification index, known as G-SI. In a longitudinal study of baboons, the authors found that approximately 95% of variations in the degree of gyrification among subjects could be attributed to growth in sulcal length and depth (Kochunov et al. 2012). Recently, a study has demonstrated that sulcal MD in the left ventrolateral prefrontal cortex can significantly affect the performance of participants in working memory related tasks, which may be due to the presence of short-range white matter fibers in the sulcal fundus (Yao et al. 2023). Accordingly, differences in sulcal MD may affect multiple brain functions, resulting in behavioral variability. Notably, a meta-analysis of 284 studies revealed that women exhibit a slight yet statistically significant superiority in verbal working memory, aligning with the results of our study (Voyer et al. 2021). Ingalhalikar et al. discovered that males demonstrate superior intrahemispheric connectivity, resulting in enhanced coordination and motor abilities. Conversely, females exhibit superior interhemispheric connectivity, facilitating better integration of diverse functions across the left and right hemispheres. This phenomenon leads to heightened language and verbal memory capabilities, as well as improved attention (Ingalhalikar et al. 2014). In terms of cognitive control, Schulte et al. found that during adolescence, males predominantly engage the extrastriate region to regulate motor control, whereas females activate this region for cognitive control. The authors attribute this divergence to the delayed maturation of males during puberty and the impact of sex hormones on brain functioning(Cservenka et al. 2015; Schulte et al. 2020).

### Limitations and Future Directions

The morphological classification of brain sulci is a process that involves subjective judgment. Despite the implementation of various measures, it is difficult to completely eliminate the influence of subjectivity. In our paper, we discussed the mechanisms underlying the establishment of the morphology-function relationship in IPCS and its functions. Future research aims to establish a direct correlation between IPCS morphology and corresponding brain functionality by comparing the behavioral patterns of individuals with different IPCS types. Moreover, investigating the association between sulcal morphology and diseases presents a viable avenue for research. In recent studies, alterations in the morphology of brain sulci have been identified in various diseases, including Alzheimer’s disease (AD) (Liu et al. 2012; Plocharski and Lasse Riis Ostergaard 2016; Mateos et al. 2020), epilepsy (Kim et al. 2008), Parkinson’s disease (PD) (Wang et al. 2021), schizophrenia (Janssen et al. 2014), coronary heart disease (Morton et al. 2020), and developmental dyslexia (Im et al. 2016). For instance, individuals diagnosed with schizophrenia display a notable reduction in cortical thickness alongside an observable enlargement in sulcal width (Janssen et al. 2014). Similarly, children with developmental dyslexia present altered patterns of sulci within specific regions, including the left parietal-temporal and occipito-temporal areas, directly associated with a decline in reading proficiency (Im et al. 2016). Sulcal parameters possess immense potential for the diagnosis of diseases and the prediction of disease prognosis. One disease that has received considerable research attention in terms of diagnostic exploration is Alzheimer’s disease. Patients with AD show widening and deepening of cerebral sulci, thinning of the cerebral cortex, and a decrease in g-SI. Notably, more pronounced changes within these cerebral sulci correspond to lower scores on the Mini-Mental State Examination (MMSE) among patients (Liu et al. 2012; Plocharski and Lasse Riis Ostergaard 2016; Morton et al. 2020). Bertoux et al. proposed that the width of cerebral sulci holds substantial diagnostic accuracy for AD. This indicator is directly associated with cognitive impairment in patients, while bearing no relationship to amyloid protein load (Bertoux et al. 2019). According to Cai et al., the measurements of g-SI and the width of the lateral fissure demonstrate high sensitivity as indicators for assessing early-stage Alzheimer’s disease (Cai et al. 2017). Furthermore, a study conducted on patients with Parkinson’s disease revealed a broad reduction in cerebral sulcal depth throughout multiple brain regions. More specifically, the decrease in cerebral sulcal depth within the left pars opercularis demonstrated a direct correlation with the decline in patients’ MMSE scores (Wang et al. 2021). This finding underscores the potential of measuring cerebral sulcal depth as a diagnostic tool for Parkinson’s disease. Particularly, the depth of cerebral sulci in the left pars opercularis can also serve as a predictor of patients’ cognitive ability in the future. Considering the role of the surrounding brain area of IPCS in human cognition and movement, there is a potential association between IPCS and disorders that lead to related functional impairments. Additionally, the sample included in this paper comprises solely of young individuals. Conducting research on the elderly population would help to reveal the influence of age on sulcal morphology.

## Conclusions

This study provides a detailed description of the morphology of IPCS and its spatial relationship with IFS, revealing the asymmetry and sexual dimorphism of IPCS. This finding will enhance our accuracy in identifying brain sulci. Building upon this foundation, it will be easier to research the effects of diseases and age on brain sulci, as well as the correlation between brain sulcal morphology and function.

## Data Availability

The datasets generated and analyzed during the current study are available from the corresponding author on reasonable request.
